# Finding Stable Graphene Conformations from Pull and Release Experiments with Molecular Dynamics

**DOI:** 10.1038/srep42356

**Published:** 2017-02-14

**Authors:** Ruslan D. Yamaletdinov, Yuriy V. Pershin

**Affiliations:** 1 Nikolaev Institute of Inorganic Chemistry SB RAS, Novosibirsk 630090, Russia; 2 Novosibirsk State University, Novosibirsk 630090, Russia; 3 Department of Physics and Astronomy and Smart State Center for Experimental Nanoscale Physics, University of South Carolina, Columbia, South Carolina 29208, USA

## Abstract

Here, we demonstrate that stable conformations of graphene nanoribbons can be identified using pull and release experiments, when the stretching force applied to a single-layer graphene nanoribbon is suddenly removed. As it is follows from our numerical experiments performed by means of molecular dynamics simulations, in such experiments, favorable conditions for the creation of folded structures exist. Importantly, at finite temperatures, the process of folding is probabilistic. We have calculated the transition probabilities to folded conformations for a graphene nanoribbon of a selected size. Moreover, the ground state conformation has been identified and it is shown that its type is dependent on the nanoribbon length. We anticipate that the suggested pull and release approach to graphene folding may find applications in the theoretical studies and fabrication of emergent materials and their structures.

There has been tremendous interest in the development of graphene-based electronics^1^ with demonstrated device prototypes ranging from the graphene transistors operating in the hundreds of gigahertz range^2^,^3^,^4^,^5^ to graphene-based nonvolatile memories^6^,^7^,^8^. While the main attention has been historically focused on the single layer graphene, its two-, three- and higher-layer varieties have been also attracting much attention. Frequently, the multilayer graphene is produced using the chemical vapour deposition on the surfaces of metals with a high carbon solubility, such as Ni^9^. A low-carbone solubility metals, such as Cu, are suitable for growth of monolayer and bilayer graphene^10^,^11^. As multilayer graphene is composed of monolayer sheets, it is tempting to explore bottom-up approaches of its fabrication, in particular, the approaches involving a controllable stacking or folding of monolayer graphene.

In this Letter, we explore various stable conformation of a graphene nanoribbon and, at the same time, demonstrate that the graphene folding can be realized in pull and release experiments. The main idea of such experiment, which is implemented here by a numerical modelling, is illustrated schematically in Fig. 1. This illustration shows three major steps of the proposed process: (i) graphene stretching by an external force, (ii) non-equilibrium dynamics after the force removal, and (iii) spontaneous emergence of a folded structure. Using molecular dynamics simulations we have demonstrated that, indeed, the process exhibited in Fig. 1 folds flat graphene nanoribbons into various stable conformations. We have classified these conformations and analyzed the probabilities of their appearance as a function of the pulling force.

Importantly, we have found that the ground state of nanoribbons depends on their length. As it is shown below, the lowest energy state of very short nanoribbons is the flat one. As the nanoribbon length increases, the energy of a bilayer (wrinkle-like) conformation becomes the lowest, and, at longer lengths, a spiral (rolled) conformation turns into the ground state. Moreover, we emphasize that a significant role in the folding process is played by the thermal fluctuations. It has been found that distinct folded states are generated in separate numerical experiments employing identical modeling parameters.

Physically, the folding occurs when the elastic potential energy of the stretched graphene is sufficient to overcome energy barriers separating the flat graphene from its folded conformations. In this way (as a part of its dynamics), the graphene may reach certain crumpled states^12^ that further develop into stable folded conformations. It should be emphasized that the process of folding by itself is not a simple process. According to our observations, the folded structure emerges through a continuous evolution of a locally nucleated bilayer graphene. Unfortunately, the dynamics of such a process is not accessible analytically. Therefore, we have performed extensive numerical simulations with a goal of finding distinctive folded states, and determining the probabilities of their formation at various values of the pulling force and types of boundary conditions.

This paper is organized as follows. In Sec. “Calculation details” we introduce the main modeling approach. Sec. “Results” presents the simulation results found in calculations with a fixed nanoribbon edge (Sec. “Fixed edge calculations”) and without fixing any atoms (Sec. “Free boundary calculations”). Moreover, the lowest energy conformation (the ground state) is investigated in Sec. “The ground state”. Finally, in Sec. “Conclusion” we report our conclusions.

## Calculation Details

We investigated the dynamics of graphene nanoribbons in pull and release experiments using classical molecular dynamics (MD) simulations. MD simulations are a well established modeling tool frequently employed in studies of nanoscale carbon-based materials^13^,^14^,^15^,^16^,^17^,^18^,^19^,^20^,^21^,^22^,^23^,^24^,^25^,^26^,^27^,^28^,^29^,^30^,^31^,^32^,^33^ and many other systems. The model system used in this work is a zigzag graphene nanoribbon of *L* = 200 Å length and *w* = 40 Å width. Two types of boundary conditions were employed. In one part of calculations, the carbon atoms at one nanoribbon edge were fixed and the stretching force was applied to the atoms at the opposite edge. In the rest of calculations, the stretching forces were applied to a pair of opposite edges without fixing any atoms. In both cases we have observed the formation of folded graphene.

MD simulations were carried out with the NAMD2 package^34^ (NAMD was developed by the Theoretical and Computational Biophysics Group in the Beckman Institute for Advanced Science and Technology at the University of Illinois at Urbana-Champaign). To describe the interactions between the carbon atoms we employed parameters of CA type atoms from CHARMM27 force field^35^,^36^. A 1 fs time step was used and the system temperature is kept at room temperature with a Langevin dampening parameter of 0.2 ps^−1^ in the equations of motion. The van der Waals interactions were gradually cut off starting at 10 Å from the atom until reaching zero interaction 12 Å away. In each simulation, the system was equilibrated for 90 ps in the presence of the pulling force and its subsequent dynamics (after the force removal) was typically simulated for 0.2–1 ns. The energy was finally minimized in 50000 time steps in order to identify precisely the energy and morphology of the final conformation.

**Figure 2:**
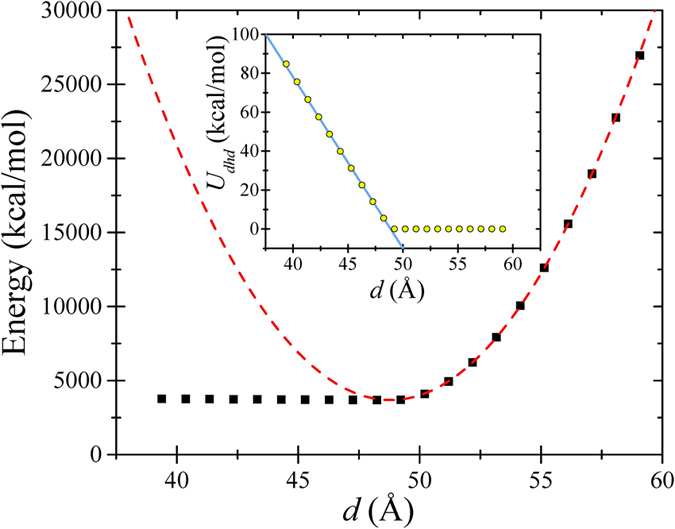
Calculated energy of a graphene nanoribbon as a function of the distance *d* between its fixed edges. Here, almost a linear dependence (the inset shows the dihedral energy contribution, *U*_*dhd*_) changes to parabolic one when *d* passes the free nanoribbon length *L* (note that at *d* = *L* the compression changes to stretching). The dashed and solid (the inset) lines are fitting curves used to extract elastic constants.

In order to validate the selected MD force field parameters, we calculated the energy of a shorter zigzag graphene nanoribbon (*L* = 49.2 Å, *w* = 40 Å) varying the distance *d* between its two opposite edges, which were kept fixed. Figure 2 shows that in the case of the stretched graphene (*d* > *L*), the energy increases quadratically with *d* to a good approximation. When *d* < *L*, a linear fit is quite good. Based on these quadratic and linear approximations we find that the in-plane stiffness *E*_2*D*_ is 380 N/m and the bending rigidity *D* is 1.93 eV. These values are in agreement with results of various experiments and calculations^37^. We thus conclude that the elastic properties of graphene nanoribbons are pretty well described by the chosen MD force field parameters. In what follows, these parameters are used without any adjustments.

## Results

### Fixed edge calculations

**Figure 3:**
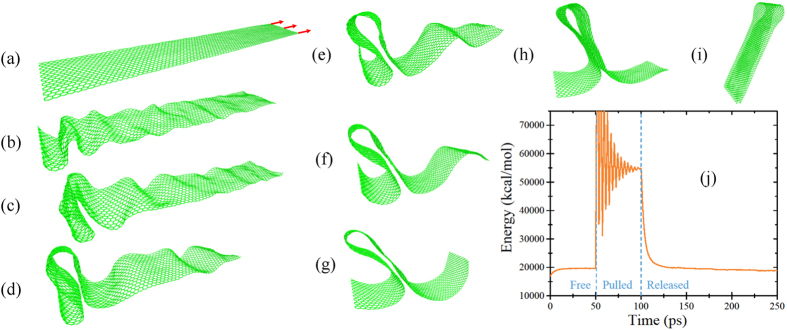
Molecular dynamics snapshots exemplifying the graphene folding process. The snapshots are shown in the increasing time order; the position of the left edge of the nanoribbon was fixed in this calculation. The pulling force is schematically shown by the red arrows. (**a**) Graphene nanoribbon stretched by an external force of 9.9 nN/atom applied to 19 atoms on the right edge. (**b**–**h**) Intermediate steps of nanoribbon dynamics corresponding to *t* = 102, 104, 107, 111, 118, 126, 136 ps in (**j**). (**i**) The final state of nanoribbon. (**j**) Nanoribbon energy as a function of time.

In the calculations presented in this Section, atoms at one nanoribbon edge were kept fixed and the pulling force was applied to 19 opposite edge atoms. An example of a run is shown in Fig. 3. This plot demonstrates that when the pulling force (applied to the right edge of nanoribbon, see Fig. 3(a)) is released, the nanoribbon center of mass starts displacing to the left (toward the fixed edge). Within this process, the nanoribbon gets crumpled in the region close to the fixed edge (Fig. 3(b,c)). Importantly, the crumpled configuration contains local nucleation spots of bilayer graphene that may “glue” different parts of graphene to each other. The final shape found in this particular calculation (Fig. 3(i)) has the structure of the graphene wrinkle, which has been studied in the literature^38^,^39^,^40^,^41^. The stacking in its bilayer region resembles the shifted stacking (see, e.g., ref. 42). The time dependence of the nanoribbon energy in the entire MD simulation is presented in Fig. 3(j).

Overall, the emergence of stable conformations from crumpled nanoribbon is quite a complex process with a high degree of randomness introduced by thermal fluctuations. In particular, we have found that, typically, the results of identical calculations can not be precisely predicted and thus should be described by a finite set of probabilities. It should be noted that the molecular dynamics reaches the stable conformations automatically since they correspond to local minima of the potential energy surface. For instance, one can notice that the final state presented in Fig. 3(i) is perfectly symmetric (the opposite sides of nanoribbon are ideally aligned) since such a symmetric shape minimizes the nanoribbon energy (note that this simulation did not include a final minimization).

**Figure 4:**
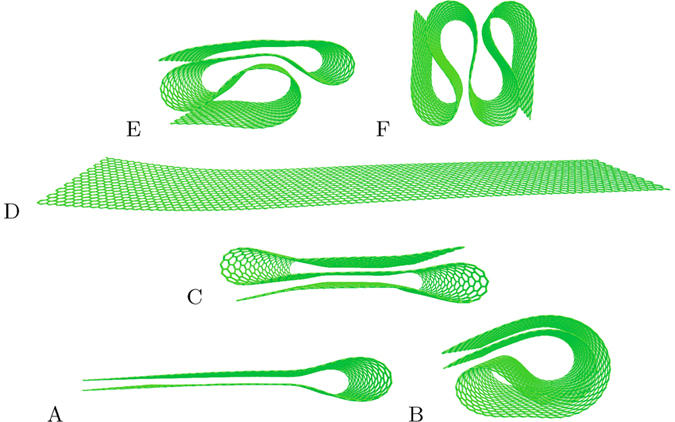
Frequently observed conformations of the nanoribbon. The conformation energy increases with the letter’s position in the alphabet.

**Figure 5:**
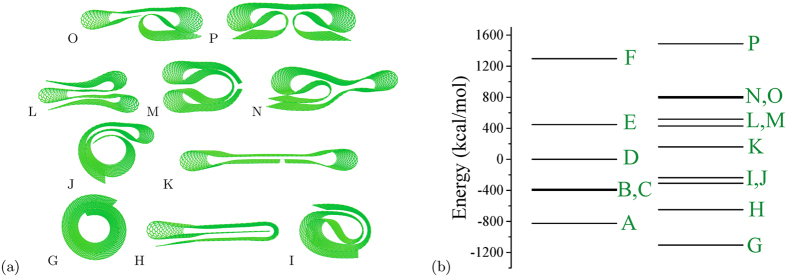
(**a**) Occasionally observed conformations of the nanoribbon. The conformation energy increases with the letter’s position in the alphabet. (**b**) Energies of nanoribbon conformations.

Altogether, we performed about 2000 numerical experiments of nanoribbon dynamics. As results of these experiments, we found stable nanoribbon conformations that can be classified according to the frequency of their appearance. It is convenient to introduce two types of conformations: frequently observed (frequent) and occasionally observed (occasional). By our convention, a conformation is frequent if it emerges with a probability >10% at least at one value of the pulling force applied (the probability distributions calculations discussed at the end of this section were used to determine and differentiate the frequent and occasional conformations). The frequent conformations are summarized in Fig. 4. In this figure (as well as in Fig. 5 presenting the occasional conformations), the conformation energy increases from bottom to top and from left to right. The conformation D is the flat nanoribbon. The shapes A and C represent pleated nanoribbon with one and two folds, respectively. In fact, one can expect the formation of pleated graphene as the most probable consequence of the nanoribbon relaxation dynamics (such as the one shown in Fig. 3). Moreover, B can be considered as a twisted variety of A.

The occasionally observed conformations are collected in Fig. 5. We should note that the shape G (the Archimedean spiral) was not spotted in our pull and release MD simulations. Here, we include it rather for the sake of completeness as the ground state of our nanoribbon (see Sec. “The ground state” for more details). At the same time, the motives of G can be seen in I and J. However, we did not observe the decay of I or J into G in longer runs, where the possibility of such decay was studied separately from our main calculations. Moreover, in Figs 4 and 5 one can notice a similarity between A and L (and also C and H). As, overall, the shapes presented in Fig. 5 are more complex than the shapes presented in Fig. 4, their formation involves more peculiar dynamics, which appears to be realized in a smaller fraction of runs.

**Figure 6:**
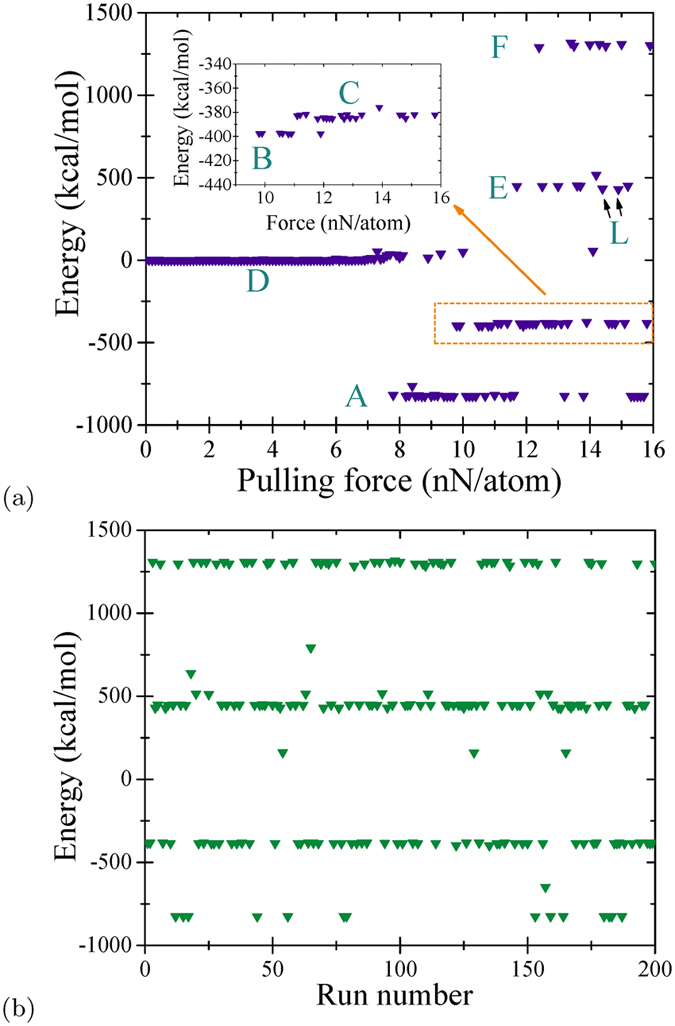
(**a**) Energy of the final conformation as a function of the pulling force in fixed-edge calculations. This plot was obtained in a single scan over the force from 0 to 16 nN per atom with 0.1 nN step. (**b**) Results of 200 identical calculations performed using the same pulling force of 14 nN per atom.

General tendencies in the formation of folded states can be seen in Fig. 6(a) that presents the final state energy (relative to the energy of the flat nanoribbon) as a function of the pulling force. In order to obtain this plot, we performed 160 runs with a pulling force changing from 0 to 16 nN/atom with 0.1 nN step. First of all we note that in Fig. 6(a) the majority of points form lines of well defined energies that we have linked to the shapes in Figs 4 and 5 and labeled accordingly. Additionally, one can notice a small degree of broadening related to slightly different energy minimization results obtained in different runs. While the energies of B and C are quite close, they can be distinguished under a close examination (Fig. 6(a)). The same is true for the energies of E and L. We have verified that each point in Fig. 6(a) is uniquely related to one of the conformations collected in Figs 4 and 5.

As it follows from Fig. 6(a), the folded states start emerging at pulling forces ~8 nN/atom. At these values of force, only one non-trivial shape (the bilayer A) is seen in the calculation results. As the force increases, the conformations B, C, E, F and L appear. Importantly, the data depicted in Fig. 5 can not be described by a sectionally continuous function. The strong stochastic component noticeable in this plot can be explained by a significant role of thermal fluctuations in the folding process.

To better understand the stochastic component in our simulation results, we performed 200 runs with the pulling force of 14 nN/atom using identical simulation parameters. The results of these runs are presented in Fig. 6(b) that shows that at this selected value of force, several frequent conformations are frequently realized. Additionally, one can spot several separate dots related to occasional conformations (or frequent conformations that are realized occasionally at 14 nN/atom pulling force). In rare cases (not in Fig. 6(b)), we also observed some not fully developed stable conformations. These not fully developed shapes can be uniquely related to the shapes collected in Figs 4 and 5. Their fraction decreases with evolution time.

**Figure 7:**
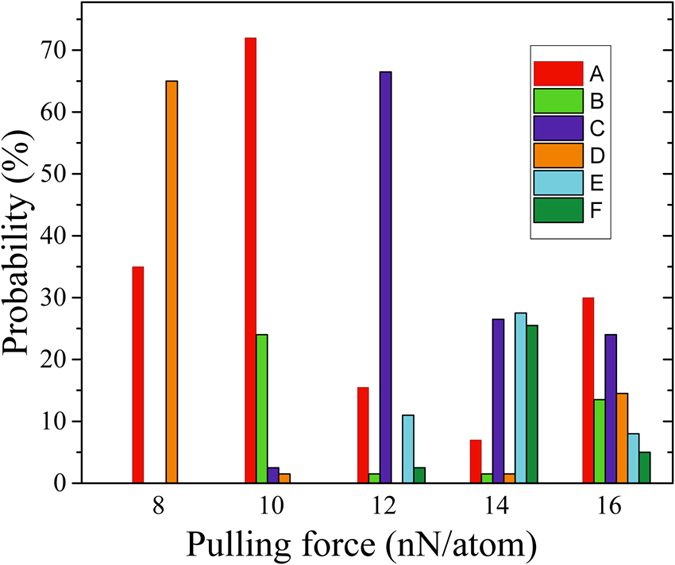
Probability distributions of final states calculated for several values of the pulling force (200 runs for each force value). Here, only the probabilities of observing the frequent conformations A–F (Fig. 4) are shown.

It seems intuitively clear that the fraction of higher-energy conformations increases with the pulling force. Indeed, in Fig. 7 this trend is well observed for the pulling forces up to 14 nN/atom. At 16 nN/atom, however, the probability distribution is shifted back (toward lower energy conformations) compared to the distribution calculated at 14 nN/atom force. As the initial kinetic energy depends on the pulling force strength, one can indeed expect the following form distributions. At smaller forces, only simple low-energy shapes are created. More conformations appear as the force increases. At larger forces, the least stable conformations start to decay (due to excessive kinetic energy allowing overcoming energy barriers separating higher energy conformations from the lower ones) increasing the fraction of simple conformations. This hypothesis provides a qualitative description of Fig. 7 results. However, future studies are needed to support it. In any case, Fig. 7 clearly shows the strong dependence of probability distributions on the pulling force.

### Free boundary calculations

Next we report on results of MD simulations performed without fixing any atoms. The pulling forces were applied to two opposite edges of the nanoribbon and released simultaneously. In these calculations, we have not observed any new conformations compared to these already presented in Figs 4 and 5. At the same time, it should be noted that the nanoribbon dynamics with free edges is different from the fixed-edge dynamics. Clearly, in the former case the center of mass does not move, so that the nanoribbon crumples near its center. In the latter case (the fixed-edge dynamics), the crumpling occurs near the fixed edge.

**Figure 8:**
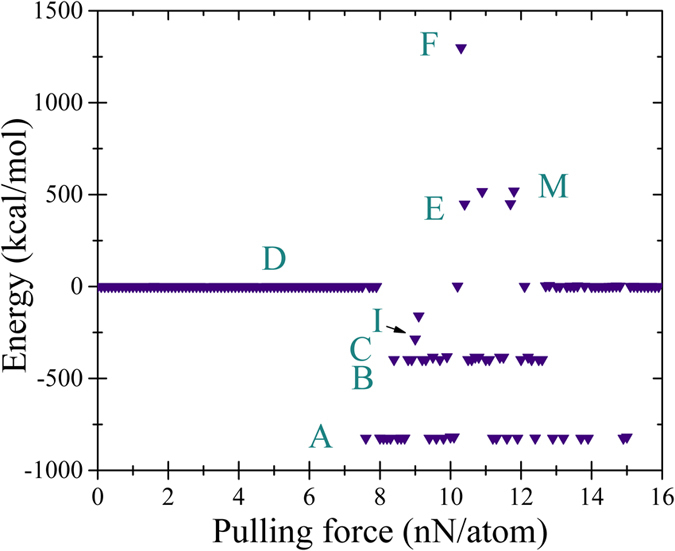
Energy of the final conformation as a function of the pulling forces applied to a pair of opposite boundaries. In this calculation, all atoms were free to move. This plot was obtained in a single scan over the force from 0 to 16 nN per atom with 0.1 nN step.

In Fig. 8 we plot the energies of final conformations found in 160 runs with the pulling force changing from 0 to 16 nN/atom in 0.1 nN step. Comparing Fig. 8 with Fig. 6(a) one can notice that in both cases the folded graphene starts emerging at the same pulling force of ~8 nN/atom. Moreover, both graphs exhibit a strong stochastic component. At the same time, the dependencies shown in Figs 6(a) and 8 are different at larger pulling forces. In this limit, the dominant final conformation in Fig. 8 is the flat one (D), unlike the result presented in Fig. 6(a) (see also Fig. 7).

### The ground state

Generally, the identification of the ground state is of a paramount importance. Based solely on the results of our MD simulations, one may think that the ground state of our nanoribbon is the conformation A. Instead, we have found that the ground state of our structure is G. The conformation G has never been observed in our MD simulations. We explain this by (i) very long times required to reach this state through the stochastic dynamics from a final conformation generated in our pull and release experiments, and (ii) zero/very small angular momentum of the system. To study the ground state, the spiral G was generated from scratch and its energy was found using an equilibration-minimization calculation.

In order to gain a better understand of the ground state of nanoribbons, below, we compare the energies of A and G without constraining the nanoribbon length to *L* = 200 Å. An approximate expression for the energy of A can be written as ref. 43


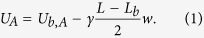


Here, *U*_*b,A*_ is the energy of U-bent region (for an expression for *U*_*b,A*_ see, for instance, Eq. (15) in ref. 43), *γ* is the adhesion energy per unit area, *L* is the total length of nanoribbon, *L*_*b*_ is the length of the U-bent region, and *w* is the nanoribbon width (in the direction along the fold).

Next, we derive an expression for the energy of G. In cylindrical coordinates, the Archimedean spiral is described by *ρ* = (*d*/(2*π*))*φ*, where *d* is the separation distance between successive turns. Let us assume that G starts at *φ* = *φ*_0_, which corresponds to the distance *R*_0_ = *φ*_0_*d*/(2*π*) from the center, and ends at a certain *φ*_*L*_. Then, one can find the following relation between the nanoribbon length *L, φ*_0_ and *φ*_*L*_:


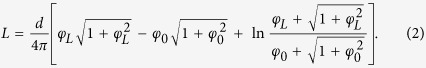


The bending energy of G shape, *U*_*b,G*_, is proportional to the integral of the squared curvature, *κ*^2^, along the spiral. Using 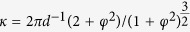, one finds


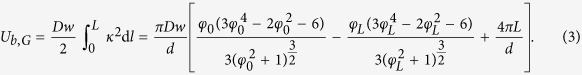


We note that a similar expression for bending energy was obtained in ref. 37. The adhesion energy of G can be written in the same form as in Eq. (1) with an effective length *L*_*eff*_ that corresponds to *φ*_*eff*_ = *φ*_*L*_ − 2*π*. The value of *L*_*eff*_ can be obtained using Eq. (2). Thus, the energy of G is given by


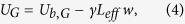


and the optimal value of *R*_0_ (corresponding to the minima of *U*_*G*_) is obtained solving the equation d*U*_*G*_/d*R*_0_ = 0.

**Figure 9:**
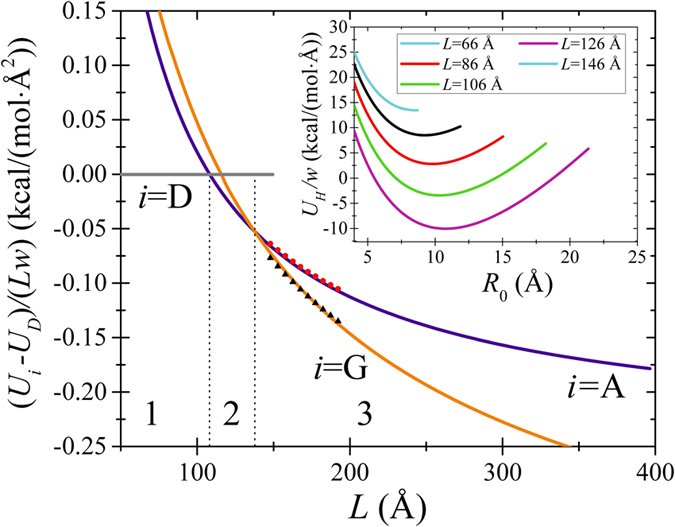
Energies of A and G as functions of *L* found using Eqs (1) and (4). The dots and triangles represent MD simulations results. Inset: dependence of the spiral conformation energy on the initial radius *R*_0_ (the shortest distance from the spiral to its center). Dotted vertical lines are shown for eye guidance to visualize domains of various ground states: D in 1, A in 2, and G in 3.

As for a given value of *L* Eq. (2) can not be solved analytically, we proceed with a numerical solution. In particular, using *D* = 1.93 eV and *γ* = 23 meV/Å^2^, we have found that the equation d*U*_*G*_/d*R*_0_ = 0 has a solution when *L* ≥ 66 Å. Figure 9 presents numerically calculated *U*_*A*_(*L*) and *U*_*H*_(*L*) plotted using the above mentioned parameter values. It follows from Fig. 9 that at *L* < 108 Å the ground state is D, for 108 Å < *L* < 138 Å the ground state is A, and for *L* > 138 Å the ground state is G. Moreover, *U*_*G*_ < 0 at *L* ≥ 116 Å.

Generally, one can show that in the limit of large *L*, the energy of the spiral conformation G is smaller than the energy of any N-folded state (e.g., the conformations A and C). Clearly, this does not prove that G is the ground state at large *L*-s. However, we have not been able to identify any other state with the energy lower than the energy of G in this limit. We thus are inclined to believe that G is the ground state of long nanoribbons.

## Conclusion

The method described here to obtain various conformations of graphene nanoribbons is quite general, straightforward, and easy to implement. It involves a stochastic component that allows the generation of distinct conformations at the same values of modeling parameters. While we have developed this approach rather as a computational tool, potentially, it can be employed in real experiments, although suitable methods to stretch and release the nanoribbon still need to be identified and developed.

## Additional Information

**How to cite this article:** Yamaletdinov, R. D. and Pershin, Y. V. Finding Stable Graphene Conformations from Pull and Release Experiments with Molecular Dynamics. *Sci. Rep.* **7**, 42356; doi: 10.1038/srep42356 (2017).

**Publisher's note:** Springer Nature remains neutral with regard to jurisdictional claims in published maps and institutional affiliations.

## Figures and Tables

**Figure 1:**
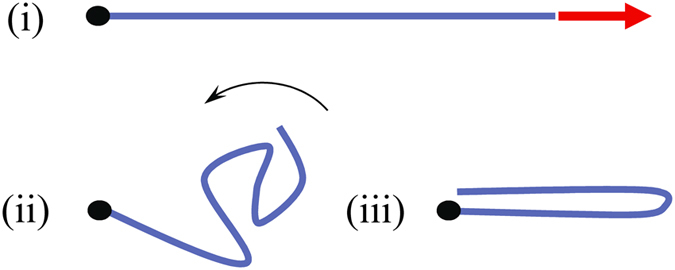
Three steps of the graphene folding mechanism studied in this work: (i) stretching, (ii) evolution after the force removal, and (iii) possible emergence of a multi-layer conformation (the final state). Here, the red arrow represents the external pulling force and the black dot represents the fixed boundary.
