# Erratum: Finding Stable Graphene Conformations from Pull and Release Experiments with Molecular Dynamics

**DOI:** 10.1038/srep44630

**Published:** 2017-03-23

**Authors:** Ruslan D. Yamaletdinov, Yuriy V. Pershin

Scientific Reports 7: Article number: 4235610.1038/srep42356; published online: 02 14 2017; updated: 03 23 2017

This Article contains an error in the Acknowledgements section.

“This work has been supported by the Russian Scientific Foundation grant No. 15-13-20021”.

should read:

“This work has been supported by the Russian Science Foundation grant No. 15-13-20021”.

